# Need-supportive digital sports platform and physical activity outcomes in university physical education: an intact-class quasi-experimental study based on self-determination theory

**DOI:** 10.3389/fpsyg.2026.1889324

**Published:** 2026-07-29

**Authors:** Jiacheng Feng, Qimeng Niu

**Affiliations:** Department of Physical Education, Tianjin University of Technology, Tianjin, China

**Keywords:** basic psychological needs, digital sports platform, need frustration, physical activity, self-determination theory, sport psychology, university physical education

## Abstract

**Introduction:**

Digital platforms are increasingly used in university physical education (PE), but the psychological pathways associated with platform-supported PE remain insufficiently understood. Because individual random assignment was not feasible in the authentic educational setting, this study examined post-intervention group differences and self-determination theory (SDT)-consistent associations, distinguishing need satisfaction from need frustration.

**Methods:**

An 8-week post-intervention intact-class quasi-experimental study was conducted in eight university table tennis classes. The final sample included 393 first- and second-year undergraduate students aged 18–21 years (control group, *n* = 191; platform-supported group, *n* = 202); exact individual age was not collected. The platform-supported group used a digital-intelligent sports platform providing course learning, training assistance, competition organization, peer interaction, and achievement feedback, whereas the control group received regular PE instruction. Outcomes included basic psychological need satisfaction and frustration, self-reported physical activity, perceived exercise persistence, self-rated PE performance, perceived change in exercise frequency, and daily sitting time. Robustness analyses included class-clustered standard errors, wild cluster bootstrap tests, false discovery rate correction, and propensity score weighting.

**Results:**

The platform-supported group reported higher need satisfaction, lower need frustration, higher log-transformed physical activity, greater perceived exercise persistence, higher self-rated PE performance, more favorable perceived change in exercise frequency, and lower daily sitting time. Cross-sectional indirect-association models showed SDT-consistent pathways through increased need satisfaction and/or reduced need frustration. The direction of the primary findings remained consistent in the supplementary sensitivity analyses.

**Discussion:**

Platform-supported PE may be understood as a motivational environment rather than merely a technological tool. However, because of non-random allocation, baseline imbalance, reliance on self-report outcomes, absence of complete pretests, and lack of objective individual-level platform usage logs, the findings should be interpreted as post-intervention group differences and cross-sectional associations rather than causal intervention effects.

## 1 Introduction

University is a critical period for the development of exercise habits, the consolidation of health-related behaviors, and the formation of lifelong physical activity awareness (Wang et al., 2025). University physical education (PE) is expected not only to teach motor skills and promote physical fitness, but also to contribute to students' long-term healthy lifestyles. However, a persistent problem in university PE is that students may complete required learning tasks in class while showing insufficient autonomous exercise outside class, weak exercise persistence, and continued dependence on external requirements. In other words, participation in PE courses does not automatically translate into sustained physical activity. How to extend PE learning from classroom tasks to daily practice and how to transform short-term course participation into more stable physical activity habits remain central challenges for university PE reform (Tao and Yu, 2025).

The rapid development of digital technologies provides new possibilities for addressing this problem. Mobile exercise applications, intelligent teaching platforms, activity-tracking tools, and artificial intelligence (AI)-assisted systems have increasingly entered PE and physical activity promotion (Liang and Wang, 2026; Yang et al., 2020). These technologies can extend learning beyond classroom time and space and provide students with sport knowledge, task reminders, data feedback, exercise records, social interaction, and competition organization. In particular, as intelligent feedback, learning analytics, and AI-assisted teaching continue to develop, technological support in PE is shifting from simply recording whether students have completed tasks to explaining how students learn, practice, and improve (Hsia et al., 2025). This shift is particularly important for PE because sport learning involves not only knowledge acquisition or conceptual understanding but also motor control, bodily perception, tactical judgment, and situated decision making. Students therefore need not only online resources, but also continuous support that connects movement knowledge, practice processes, feedback suggestions, and peer contexts.

Nevertheless, technology use does not necessarily lead to positive behavioral outcomes. If a digital platform is used only for monitoring, check-ins, rankings, or external control, students may merely complete assigned tasks passively without developing stable physical activity engagement (Kidman et al., 2024). Conversely, if a platform provides understandable practice pathways, actionable feedback, sustainable competition opportunities, and perceived peer support, it may become a more educationally meaningful physical activity environment. Thus, research on digital-intelligent sports platforms should not only ask whether students use a platform, but should also examine under what psychological conditions the platform is associated with students' physical activity outcomes.

Self-determination theory (SDT) provides an important theoretical basis for understanding the relationship between digital sports platforms and student physical activity engagement. SDT argues that sustained participation in an activity depends not merely on external demands or rewards, but on whether the activity context supports the basic psychological needs for autonomy, competence, and relatedness (Deci and Ryan, 2000). Autonomy refers to feeling that one's behavior is volitional and self-endorsed; competence refers to feeling effective, capable of improvement, and able to meet challenges; relatedness refers to feeling supported, understood, and connected with others. When these basic psychological needs are satisfied, individuals are more likely to experience high-quality engagement and to show greater involvement, persistence, and behavioral maintenance; when these needs are frustrated, individuals may experience pressure, incompetence, or alienation, which can undermine sustained participation (Ryan and Deci, 2020).

In PE, basic psychological needs have been widely used to explain students' physical activity participation, classroom engagement, exercise persistence, and perceived performance (White et al., 2021). Teacher support, classroom climate, peer interaction, and learning task structures may influence PE outcomes through students' experiences of autonomy, competence, and relatedness (Ji et al., 2025). Compared with conventional classroom support, digital-intelligent sports platforms have cross-temporal, process-oriented, and data-based characteristics that can extend teacher guidance, peer interaction, and individual feedback beyond the classroom. A digital platform may therefore constitute a new physical activity environment rather than a mere technical medium. The key issue is whether this platform environment genuinely supports students' basic psychological needs or simply reinforces external supervision and task management.

The platform examined in this study was not a general exercise-recording application, but an integrated support system embedded in university PE and everyday exercise contexts. It was designed around a data-knowledge synergy logic: sport knowledge, course tasks, practice records, match data, and peer interaction information were organized into a continuous support chain rather than isolated digital records. In this environment, students could access movement cues, project rules, practice suggestions, progress feedback, match organization, and peer communication. Theoretically, course knowledge and task choice may support students' autonomy by improving task understanding and self-regulation; feedback, learning paths, and progress visualization may support competence by making improvement more visible; and match organization and social interaction may support relatedness by embedding individual practice within peer networks.

Although intelligent systems, mobile applications, and digital platforms have increasingly been introduced into PE, the psychological contribution of this literature remains underdeveloped (Sultoni et al., 2021). First, many studies have evaluated usability, skill performance, or fitness outcomes, but have provided less insight into how digital PE environments are associated with motivational experiences. Second, existing studies rarely foreground the dual role of need satisfaction and need frustration in digital PE contexts, even though SDT suggests that supporting positive need experiences and reducing need-blocking experiences are not identical processes. Third, evidence from real university PE settings remains limited, particularly for intact-class platform-supported environments in which individual random assignment is difficult. Therefore, the present study does not aim to prove platform effectiveness in a strong causal sense; rather, it examines whether need satisfaction and need frustration show distinct SDT-consistent pathways in a platform-supported PE environment.

Based on this rationale, the present study conceptualized the digital-intelligent sports platform as a need-supportive PE environment and examined whether platform-supported conditions were associated with more favorable psychological need states and physical activity outcomes. The central psychological contribution is the separation of the positive pathway of need satisfaction from the negative pathway of need frustration. This framing shifts the evaluation of digital PE platforms from a technology-effect perspective to a motivational-environment perspective. Rather than treating platform use as a simple causal exposure variable, we examine whether the platform-supported environment is associated with students' autonomy-, competence-, and relatedness-related experiences, and whether favorable physical activity outcomes are more closely linked to enhanced positive need experiences, reduced negative need-blocking experiences, or both.

This study addressed two research questions: RQ1: Are there post-intervention differences between the platform-supported group and the control group in basic psychological need states and physical activity outcomes? RQ2: Do need satisfaction and need frustration show cross-sectional indirect associations between group membership and physical activity outcomes, thereby indicating SDT-consistent psychological pathways? Based on SDT, two hypotheses were proposed. H1: Compared with the control group, students under platform-supported conditions were expected to report higher basic psychological need satisfaction, lower basic psychological need frustration, and more favorable physical activity outcomes. H2: Increased need satisfaction and/or reduced need frustration were expected to show statistical indirect associations between group membership and physical activity outcomes.

## 2 Materials and methods

### 2.1 Theoretical framework and research model

This study was grounded in SDT and focused on the relationship between platform-supported PE, basic psychological needs, and physical activity outcomes. Rather than restating the general assumptions of SDT, the framework specified how platform functions could map onto need support. Course pathways, movement-knowledge prompts, and course review were expected to support autonomy by helping students understand tasks and regulate practice. Training plans, feedback prompts, and intelligent question-answering were expected to support competence by helping students identify problems, receive actionable guidance, and perceive progress. Competition matching, peer interaction, and achievement feedback were expected to support relatedness by embedding individual practice within peer connections and a class-based sport community.

In the research model, group membership represented the platform-supported condition, Need_Sat and Need_Fru were specified as the main psychological variables, and self-reported physical activity level, perceived exercise persistence, self-rated PE performance, perceived change in exercise frequency, and daily sitting time were specified as outcomes. Need_Comp was retained only as a supplementary composite index. Because the study used an intact-class quasi-experimental design and did not include complete pretest-posttest measures for all variables, model estimates are interpreted as statistical associations and potential psychological pathways rather than strict causal mechanisms.

### 2.2 Research design

This study adopted a post-intervention intact-class quasi-experimental design in a real university table tennis course to examine differences in students' psychological need states and physical activity outcomes under platform-supported conditions (Miller et al., 2020). Eight intact teaching classes were involved, with four classes in the platform-supported group and four in the control group. Each class included approximately 50 students. All classes were taught by the same instructor, followed the same sport project (table tennis), used the same facilities, and adopted the same assessment criteria. The intervention lasted 8 weeks, with two PE sessions per week, resulting in 16 sessions in total. Because students were organized according to existing teaching classes, individual random assignment was not feasible.

The platform-supported group used the digital-intelligent sports platform in addition to regular table tennis instruction, whereas the control group received regular PE instruction. In this study, regular PE instruction refers to PE teaching organized through classroom explanation, on-site demonstration, group practice, teacher feedback, and course assessment under the same instructor, sport project, and assessment standards. The control group did not lack teacher guidance; rather, it did not use the digital-intelligent sports platform for after-class review, match organization, training reminders, or social interaction.

Platform-supported classes were scheduled on Mondays and Tuesdays, whereas control classes were scheduled on Thursdays and Fridays. Class periods included 08:00–09:30, 10:00–11:30, 14:00–15:30, and 16:00–17:30. During implementation, the two groups were consistent in instructor, sport project, teaching venue, assessment approach, and evaluation criteria. Because the study was conducted within existing classes and course schedules, students were not randomly assigned at the individual level. Therefore, this study should be interpreted as an intact-class quasi-experiment rather than a randomized controlled trial, and weekday scheduling differences are treated as a potential source of confounding.

The instructor had experience teaching university table tennis and completed platform function training before the study. The instructor was familiar with the teacher dashboard, course guidance, competition organization, and student feedback functions. Students in the platform-supported group had not systematically used the platform before the study. During the first class, the instructor introduced platform login procedures, core functions, and basic operations to reduce the influence of initial differences in platform familiarity on course implementation. The same course objectives, movement requirements, class assessment rubrics, and classroom practice structure were used across all classes to reduce implementation variation unrelated to platform support.

### 2.3 Participants, grouping, and ethics

Participants were recruited from eight intact teaching classes in a university public PE table tennis course. The participants were first- and second-year undergraduate students aged 18–21 years; exact individual age was not collected in the questionnaire and therefore could not be summarized as a mean or included as a covariate. The theoretical focus of this study was not university students as a population *per se*, but the relationships among platform-supported conditions, basic psychological need states, and physical activity outcomes. The university student sample served as the educational context and empirical carrier for testing these relationships.

The final valid sample included 393 students, with 191 in the control group and 202 in the platform-supported group. Group information was coded by the researchers according to teaching class. Both groups completed the same core questionnaire. No platform satisfaction or platform experience items were included in the core questionnaire, thereby preserving measurement equivalence across groups.

Before implementation, participants were informed of the study purpose, questionnaire content, data use, and anonymous data processing procedures. Students participated voluntarily and could withdraw at any time; participation did not affect course grades or teacher evaluation. All data were anonymized and used only for academic research. The study was reviewed and approved by the Science and Technology Ethics Committee of Tianjin University of Technology (approval no. TUT-R-202603270029).

Because the study used an intact-class quasi-experimental design, participants were not randomly allocated to conditions. Therefore, group differences in demographic characteristics and baseline exercise frequency were possible. Baseline characteristics and between-group comparability are summarized in Table 1. To improve interpretive rigor, baseline exercise frequency was controlled as a covariate in regression and indirect-association models, and demographic variables were included in robustness models. These statistical controls were used to reduce observable imbalance, but they cannot replace individual random assignment or complete pretest measurement.

**Table 1:** Baseline characteristics by group.

Characteristic	Control (*n* = 191)	Platform-supported (n=202)	Test	Statistic	*p*
Gender			Chi-square test	22.722	< 0.001
Female	49 (25.7%)	100 (49.5%)			
Male	142 (74.3%)	102 (50.5%)			
Grade			Chi-square test	3.733	0.053
First-year	65 (34.0%)	89 (44.1%)			
Second-year	126 (66.0%)	113 (55.9%)			
Academic major			Chi-square test	102.818	< 0.001
Education, arts, or physical education	25 (13.1%)	64 (31.7%)			
Humanities	15 (7.9%)	48 (23.8%)			
Social sciences	24 (12.6%)	57 (28.2%)			
Science, engineering, agriculture, or medicine	127 (66.5%)	33 (16.3%)			
Baseline exercise frequency	3.25 (0.83)	2.03 (0.70)	Welch *t* test	*t* = −15.569; d = −1.579	< 0.001

Values for categorical variables are *n* (%). Values for baseline exercise frequency are *M* (SD). Chi-square tests were used for categorical variables unless Fisher's exact test was required because of small expected cell counts. Welch *t* tests were used for continuous variables. Cohen's d was calculated as the standardized mean difference, with positive values indicating higher values in the platform-supported group.

### 2.4 Digital-intelligent sports platform intervention

**Figure 1:**
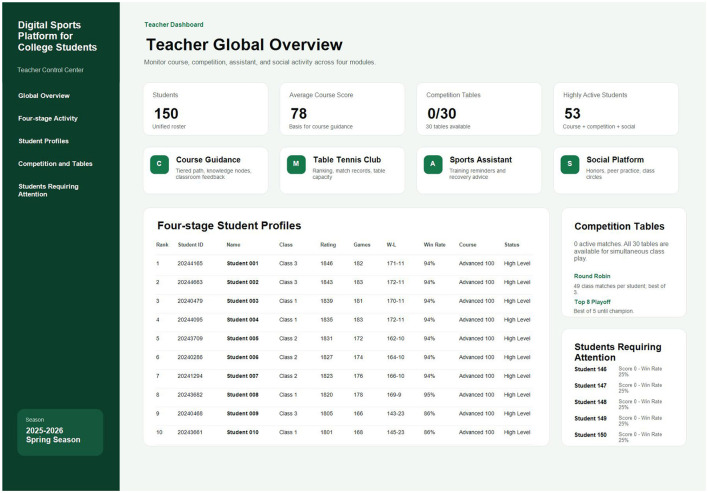
De-identified teacher-side dashboard of the digital-intelligent sports platform. The dashboard allowed the instructor to organize course guidance, competition records, assistant interaction, social engagement, and student progress information in a single teacher-facing interface.

**Figure 2:**
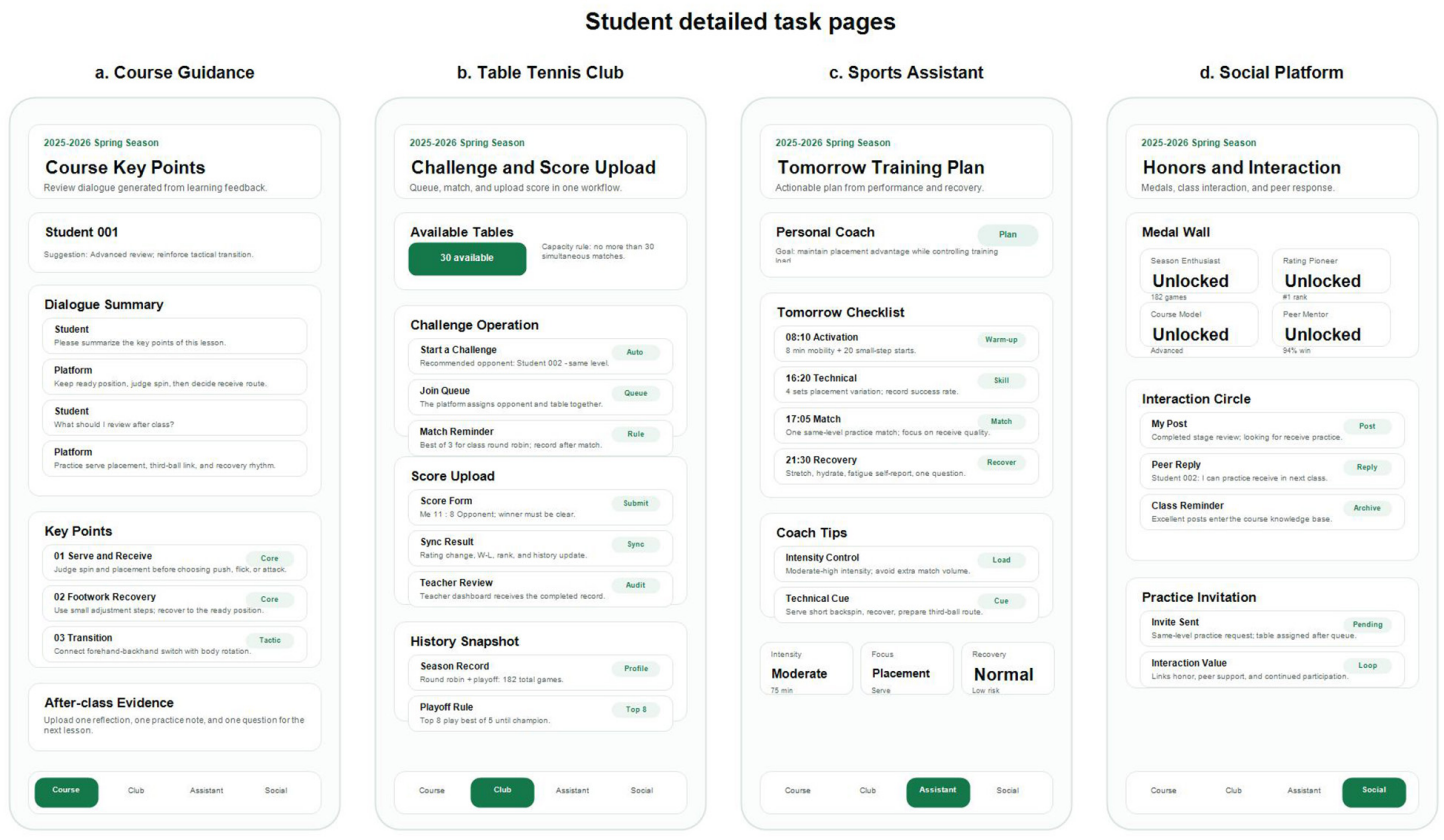
De-identified student-facing interfaces of the digital-intelligent sports platform. The panels illustrate course guidance and review functions, table tennis club competition and score-upload functions, sports-assistant training and recovery-planning functions, and peer social-interaction and achievement-feedback functions.

The platform used by the platform-supported group was designed for table tennis course learning, extracurricular practice, competition participation, and peer interaction. It adopted a teacher-side/student-side collaborative structure. The teacher side provided an overview of course guidance, student profiles, competition status, assistant interactions, and social engagement, allowing the instructor to monitor the learning process and provide organizational support. The student side provided access to course guidance, learning-path feedback, club competition, match invitation, score uploading, peer recommendation, social interaction, and achievement feedback. Representative de-identified interfaces are shown in Figures 1, 2.

Functionally, the platform consisted of four interrelated modules: course learning, training assistance, club competition, and social-achievement support. The course learning module transformed movement knowledge, stage tasks, course review, and evaluative feedback into a revisitable learning path. For example, after a class on forehand drive or footwork practice, students could review key movement cues, practice requirements, and staged learning tasks. The training assistance module provided training plans, technical tasks, intelligent Q&A, and recovery reminders based on course performance, match load, and students' learning stage; for instance, a student who reported difficulty maintaining rally consistency could receive staged practice suggestions such as shadow swing repetition, fixed-point forehand practice, or peer rally tasks. The club competition module supported match invitation, automatic matching, score uploading, rankings, and historical match records. The social-achievement module used peer recommendation, sport posts, interaction prompts, and badge feedback to strengthen participation visibility and group belonging (Goodyear et al., 2021; Looyestyn et al., 2017).

In implementation, the platform operated as a regular support condition rather than a one-time online task. In class, the instructor used platform-based course pathways, movement prompts, and competition records to organize practice and matches. After class, students could review course content, consult training suggestions, initiate matches, upload scores, and interact with peers. For example, students could invite classmates to table tennis matches, record scores, view participation records, and receive visible achievement feedback. The instructor remained responsible for learning objectives, movement standards, and assessment, while the platform mainly provided process recording, feedback prompts, and extracurricular organization.

The term digital-intelligent did not refer to a single algorithmic module. Rather, it described a support environment in which course knowledge, student performance, competition records, training reminders, and interaction feedback were organized into a continuous learning-support chain. AI-supported functions were mainly used for text-based question answering, course-content retrieval, and training-suggestion generation, rather than automated diagnosis of movement quality. Thus, the study examined a teacher-guided, platform-supported, student-engaged PE environment rather than the isolated effect of a single AI algorithm.

From an SDT perspective, platform functions corresponded to autonomy, competence, and relatedness support. Course pathways and review suggestions supported autonomy by helping students understand tasks, choose practice strategies, and regulate learning. Training plans, diagnostic prompts, and feedback suggestions supported competence by helping students identify problems, perceive progress, and receive actionable guidance. Match invitations, peer recommendation, sport communities, and badge feedback supported relatedness by connecting students with peers and the class sport community. The correspondence between platform modules, task functions, student use, teacher support, and theoretical mechanisms is summarized in Table 2.

**Table 2:** Digital-intelligent sports platform modules, task functions, and theoretical mechanisms.

Platform module	Main functions	Student use	Teacher support	Theoretical mechanism
Teacher dashboard	Student profiles, course status, competition participation, active students	—	Understand learning and participation processes; organize, remind, and provide feedback	Process-oriented support environment
Course module	Stage pathways, movement knowledge graph, course review, evaluation feedback	View movement cues, learning tasks, and review suggestions	Use platform information for classroom guidance and after-class reminders	Autonomy and competence support
Table tennis club	Points, ranking, win rate, match invitations, matching, score uploads	Initiate matches, participate in competitions, view records	Organize competitions, review records, maintain season order	Competence and relatedness support
Sports assistant	Training plans, intelligent Q&A, technical tasks, recovery reminders	View training suggestions and complete technical practice	Extend classroom requirements into after-class training prompts	Autonomy and competence support
Social and achievement module	Sport circles, peer recommendations, posts, badges	Post updates, invite practice, receive achievement feedback	Build a class-based sport community	Relatedness and competence support

Modules were summarized from the teacher-side and student-side functions of the platform.

### 2.5 Implementation fidelity and contamination control

Several procedures were used to strengthen implementation consistency and reduce contamination between conditions. First, all classes were taught by the same instructor, followed the same table tennis syllabus, and used the same facilities, class duration, assessment criteria, and course evaluation standards. Second, the platform-supported group received standardized login and operation guidance in the first class, and the instructor used the same teacher-side platform functions across the supported classes, including course guidance, competition organization, and student feedback. Third, the control group received regular PE instruction without platform-based after-class review, competition organization, training reminders, or social interaction functions. Fourth, both groups completed the same core questionnaire, and no platform satisfaction items were included in the between-group outcome measures. However, no third-party classroom observation, video coding, or independent fidelity rating was conducted, and individual-level backend use-dose data could not be exported from the platform. Therefore, implementation fidelity was monitored at the course-process level rather than through objective student-level platform-use logs; this limitation is acknowledged in the Discussion.

### 2.6 Measures

The measurement design followed four principles. First, core variables were aligned with the basic constructs of SDT, focusing on basic psychological need satisfaction and frustration rather than platform satisfaction or function evaluation. Second, the platform-supported and control groups completed the same core questionnaire with identical items, response formats, and scoring rules. Third, established scales were used through contextual wording adaptation: the theoretical structure was retained, while item contexts were adjusted to university PE, table tennis learning, and extracurricular practice. Fourth, because the study aimed to examine psychological pathways under platform-supported conditions, platform usability and satisfaction items were not included in the core between-group questionnaire to avoid creating group-specific measurement content.

Basic psychological need satisfaction and frustration were measured based on the theoretical structure of the Basic Psychological Need Satisfaction and Frustration Scale (BPNSFS) (Behzadnia, 2021). The scale is rooted in SDT and measures satisfaction and frustration of autonomy, competence, and relatedness. Measurement of psychological need satisfaction has also been widely applied in exercise contexts (Wilson et al., 2006). Compared with tools that measure only need satisfaction, BPNSFS is useful because it distinguishes positive need experiences from negative need-blocking experiences, consistent with this study's dual-path focus on increased need satisfaction and reduced need frustration.

The adapted questionnaire retained the six-dimensional structure of BPNSFS: autonomy satisfaction, autonomy frustration, competence satisfaction, competence frustration, relatedness satisfaction, and relatedness frustration. Each dimension included four items, resulting in 24 items. Items were adapted from general life contexts to PE and exercise contexts to improve interpretive consistency. For example, wording referring to general daily activities was contextualized as PE learning, table tennis practice, or extracurricular exercise, and wording referring to people around the respondent was contextualized as teachers, classmates, or peers in PE and exercise contexts. This adaptation was a contextual wording adjustment rather than a change in the dimensional structure, response scale, or scoring direction of the original scale.

All items were rated on a 5-point Likert scale ranging from 1 (strongly disagree) to 5 (strongly agree). Need satisfaction items and need frustration items were scored in their original directions, with higher scores indicating higher satisfaction or higher frustration, respectively. Total need satisfaction (Need_Sat) and total need frustration (Need_Fru) were calculated separately. A composite psychological need index (Need_Comp = Need_Sat—Need_Fru) was also constructed to supplementarily represent overall psychological need state. Because need satisfaction and need frustration are not simply opposite ends of a single continuum, Need_Sat and Need_Fru were used as primary model variables, whereas Need_Comp was used as a **supplementary** indicator.

Physical activity level was assessed using a 7-day recall self-report framework and calculated according to the International Physical Activity Questionnaire (IPAQ) scoring logic for MET-min/week at different intensities (Craig et al., 2003). Self-report measures are commonly used to assess physical activity in mobile application and intervention research (Lee et al., 2025; He et al., 2024). In this study, instructions and examples were adapted to the university PE context so that students could recall recent PE exercise, extracurricular practice, and daily physical activity.

Vigorous-intensity activity, moderate-intensity activity, and walking were calculated separately in MET-min/week and then summed to obtain MET_total. The formulas were: vigorous activity MET = 8.0 x days x daily min; moderate activity MET = 4.0 x days x daily min; walking MET = 3.3 x days x daily min. Daily sitting time (SittingMin) was reported separately as a supplementary physical activity-related indicator and was not included in MET_total. Because IPAQ data are often skewed and self-reported physical activity may be overestimated, log_MET_total was used as the primary physical activity outcome, and raw MET_total and winsorized MET_total were reported in robustness analyses (Brenner and DeLamater, 2014).

In addition to physical activity level, perceived exercise persistence (Persist), perceived change in exercise frequency (Change), and self-rated PE performance (PE_Perf) were measured as outcome variables. Persist reflected students‘ subjective tendency to maintain exercise participation; Change reflected perceived changes in exercise frequency compared with previous habits; and PE_Perf reflected students' self-evaluation of performance in PE learning and practice.

These variables were not treated as complete psychological scales; rather, they were included as self-reported outcomes directly related to the research questions. PE_Perf should not be equated with objective PE grades, Persist should not be equated with actual persistence behavior, and Change should not be equated with objectively measured frequency change. Accordingly, the terms self-reported physical activity level, perceived exercise persistence, perceived change in exercise frequency, and self-rated PE performance were used throughout the results and discussion.

Baseline exercise frequency (BaseEx) was used as the primary covariate. This variable reflected students' pre-existing physical activity foundation and helped control for the influence of prior exercise habits on psychological needs and physical activity outcomes. Given that intact-class grouping could also involve differences in gender, grade, and academic major, these demographic variables were included in robustness models, and class-clustered robust standard errors were calculated.

### 2.7 Data collection and quality control

The questionnaire was administered anonymously after the intervention. Students were informed of the study purpose, response requirements, and data use. They were told that the questionnaire was for academic research only and would not influence course grades or teacher evaluation. Both groups completed the same core questionnaire to ensure measurement equivalence for between-group comparisons.

Valid questionnaires were identified according to three principles: core scale items had to be complete; physical activity items had to be sufficient for calculating MET data; and no obvious invalid response pattern could be present. Invalid or suspicious responses were identified using rules related to long-string identical responses, extreme contradictions between need satisfaction and need frustration, ceiling responses on subjective outcomes, extreme IPAQ values, MET_total outliers, and inconsistent variable combinations. A total of 393 valid responses were included in the final analysis.

### 2.8 Data analysis

The analysis proceeded through data auditing, reliability and validity testing, descriptive statistics, baseline comparison, group difference testing, covariate-controlled regression, correlation analysis, mediation testing, and robustness analysis. First, the two datasets were checked for field consistency, missing values, outliers, and physical activity scoring rules. Full-sample data quality auditing was then conducted. Quality flags were used only to identify data quality risks and perform sensitivity analyses; original data were not modified after the fact.

Because BPNSFS items were contextually adapted to PE and exercise, Cronbach's alpha, McDonald's omega, confirmatory factor analysis (CFA) fit indices, convergent validity, and discriminant validity were examined. CFA reported chi-square/df, CFI, TLI, RMSEA, and SRMR, together with standardized factor loadings, composite reliability, average variance extracted, and latent factor correlations. Missing-data handling and outlier checks were completed before inferential analyses; no *post hoc* modification of original responses was conducted.

Group differences were examined using Welch's *t* tests, and Cohen's d was reported as the effect size (Cohen, 1988). Because effect sizes were calculated from post-intervention group differences, they were interpreted as descriptive standardized differences rather than treatment-effect estimates. For skewed physical activity variables, log_MET_total and winsorized MET_total were also reported. To address multiplicity in post-intervention group comparisons, Benjamini-Hochberg false discovery rate (FDR) correction was applied separately within two predefined families of variables: psychological-need variables and physical activity/self-reported outcome variables. Regression models were then used to examine associations between group membership and psychological need variables or physical activity outcomes after controlling for BaseEx. Robustness models additionally controlled gender, grade, and academic major and calculated robust standard errors clustered by class_id. Because students were nested within intact classes, class clustering was treated as a sensitivity issue (Preacher et al., 2011). With only eight intact classes, class-clustered robust standard errors and wild cluster bootstrap results were interpreted as sensitivity evidence rather than the sole basis of inference. As an additional sensitivity analysis for non-random allocation and observed baseline imbalance, stabilized inverse probability of treatment weighting (IPTW) was conducted using a logistic propensity score model based on gender, grade, academic major, and BaseEx. Age could not be included in the propensity score model because exact individual age was not collected. Weighted linear models with robust standard errors were used to examine whether the direction of group-outcome associations was consistent after accounting for observed baseline covariates. IPTW was interpreted as a sensitivity analysis for observed covariates and not as a method that eliminates unmeasured confounding.

Regression-based bootstrap procedures were used to estimate cross-sectional indirect associations (Hayes, 2009). Because need satisfaction and need frustration are related but distinct constructs, Need_Sat and Need_Fru were specified as the main parallel psychological variables. Need_Comp was used as a supplementary composite index. All indirect-association models controlled BaseEx, and robustness models further considered demographic variables. Bootstrap resampling was set at 5,000 draws; an indirect association was considered statistically detectable when the 95% confidence interval did not include zero. Because the psychological variables and outcomes were measured at the same post-intervention time point and complete pretest-posttest measures were not available, these models were interpreted as SDT-consistent cross-sectional indirect associations rather than causal mediation effects.

## 3 Results

### 3.1 Sample characteristics and baseline comparability

The final sample included 393 first- and second-year undergraduate students aged 18–21 years, with 191 in the control group and 202 in the platform-supported group. Exact individual age was not collected in the questionnaire, which limited demographic description and prevented age adjustment in the statistical models. As shown in Table 1, the two groups differed significantly in gender distribution (chi-square = 22.722, *p* < 0.001), academic major (chi-square = 102.818, *p* < 0.001), and baseline exercise frequency (*t* = −15.569, *p* < 0.001, *d* = −1.579), whereas the difference in grade approached but did not reach the conventional level of statistical significance (chi-square = 3.733, *p* = 0.053). To reduce the influence of pre-existing exercise habits on interpretation, subsequent regression and indirect-association models controlled BaseEx. It is noteworthy that baseline exercise frequency was lower in the platform-supported group than in the control group; therefore, more favorable psychological and physical activity outcomes in the platform-supported group cannot be simply attributed to a higher pre-existing physical activity foundation. However, these baseline imbalances remain an important limitation of the non-randomized design.

### 3.2 Reliability and structural validity

BPNSFS showed good internal consistency in this sample (Dunn et al., 2014). Cronbach's alpha values for the six subdimensions ranged from 0.753 to 0.879, and McDonald's omega values ranged from 0.766 to 0.883. The alpha and omega values for Need_Sat were 0.917 and 0.919, respectively, and those for Need_Fru were 0.912 and 0.916, respectively. Detailed reliability and structural validity information is provided in Supplementary Table 1.

To examine the structural suitability of the contextually adapted BPNSFS, a six-factor CFA model was tested. The model fit was acceptable: chi-square/df = 2.270, CFI = 0.944, TLI = 0.934, RMSEA = 0.057, and SRMR = 0.047. Model comparison results showed that the six-factor model fit the data better than two-factor, three-factor, and one-factor models, supporting the retention of the six-dimensional structure distinguishing satisfaction and frustration of autonomy, competence, and relatedness.

Regarding convergent validity, all dimensions had composite reliability values above 0.70, and most AVE values reached or approached 0.50 (Fornell and Larcker, 1981). However, the AVE values for relatedness satisfaction and relatedness frustration were slightly below the ideal threshold, suggesting that these dimensions should be interpreted cautiously. For discriminant validity, several first-order latent factors showed high correlations, indicating strong associations among the six subdimensions. Therefore, Need_Sat and Need_Fru were used as the primary theoretical dimensions in subsequent pathway analyses, while Need_Comp was retained only as a supplementary composite index.

### 3.3 Group differences

Post-intervention between-group comparisons showed that the platform-supported group reported significantly more favorable basic psychological need states than the control group. Specifically, need satisfaction, the composite psychological need index, and the three need satisfaction subdimensions were significantly higher in the platform-supported group, whereas need frustration and the three need frustration subdimensions were significantly lower. The largest descriptive standardized difference appeared in competence frustration, suggesting that platform-supported conditions were associated with lower reported feelings of incompetence, frustration, and uncertainty in PE practice. Because these comparisons were based on post-intervention data without complete pretests, the effect sizes should be interpreted as descriptive group differences rather than causal treatment effects. Detailed between-group comparisons of the psychological-need variables are presented in Table 3.

**Table 3:** Between-group differences in basic psychological need variables.

Variable	Control *M* (SD)	Platform *M* (SD)	Mean diff.	*t*	df	*p*	Cohen's *d*
Need_Sat	3.827 (0.720)	4.394 (0.286)	0.566	10.140	245.564	< 0.001	1.045
Need_Fru	2.312 (0.770)	1.506 (0.231)	−0.805	−13.882	222.119	< 0.001	−1.434
Need_Comp	1.516 (1.356)	2.887 (0.382)	1.372	13.479	218.435	< 0.001	1.393
Auto_Sat	3.877 (0.833)	4.386 (0.347)	0.509	7.828	251.197	< 0.001	0.806
Auto_Fru	2.111 (0.955)	1.520 (0.299)	−0.591	−8.191	224.949	< 0.001	−0.846
Comp_Sat	3.726 (0.826)	4.386 (0.369)	0.660	10.123	259.755	< 0.001	1.041
Comp_Fru	2.952 (0.942)	1.510 (0.310)	−1.442	−20.149	228.740	< 0.001	−2.080
Rel_Sat	3.878 (0.732)	4.408 (0.326)	0.530	9.190	259.203	< 0.001	0.945
Rel_Fru	1.872 (0.775)	1.489 (0.307)	−0.383	−6.369	245.314	< 0.001	−0.656

Mean differences were calculated as platform-supported group minus control group. Welch *t* tests were used. Need_Sat, total need satisfaction; Need_Fru, total need frustration; Need_Comp = Need_Sat – Need_Fru.

For physical activity outcomes, MET_total, log_MET_total, perceived exercise persistence, self-rated PE performance, and perceived change in exercise frequency were significantly higher in the platform-supported group than in the control group, whereas daily sitting time was significantly lower. These results indicate that the platform-supported group showed more favorable self-reported physical activity and participation outcomes, but they should not be interpreted as direct evidence of individual-level behavioral change. Detailed between-group comparisons of the physical activity and self-reported outcomes are presented in Table 4.

**Table 4:** Between-group differences in physical activity outcomes.

Variable	Control *M* (SD)	Platform *M* (SD)	Mean diff.	*t*	df	*p*	Cohen's *d*
MET_total	2,120.212 (1,547.252)	4,908.235 (862.147)	2,788.023	21.896	293.984	< 0.001	2.243
log_MET_total	7.176 (1.583)	8.483 (0.183)	1.307	11.342	194.812	< 0.001	1.176
Persist	3.393 (0.972)	4.109 (0.526)	0.716	9.012	288.822	< 0.001	0.924
PE_Perf	3.131 (0.807)	4.460 (0.519)	1.330	19.303	321.314	< 0.001	1.971
Change	3.524 (0.972)	4.124 (0.518)	0.600	7.575	286.129	< 0.001	0.777
SittingMin	413.010 (183.287)	374.777 (57.572)	−38.233	−2.757	225.251	0.006	−0.285

MET_total refers to total physical activity in the past 7 days, expressed as MET-min/week. log_MET_total, log (MET_total + 1). Persist, perceived exercise persistence; PE_Perf, self-rated PE performance; Change, perceived change in exercise frequency; SittingMin, daily sitting time.

### 3.4 Regression analysis controlling baseline exercise frequency

After controlling BaseEx, group membership remained significantly associated with psychological need variables and major physical activity outcomes. Platform-supported group membership was positively associated with need satisfaction, Need_Comp, MET_total, log_MET_total, perceived exercise persistence, self-rated PE performance, and perceived change in exercise frequency, and negatively associated with need frustration and daily sitting time. These results indicate that group differences were not fully explained by measured baseline exercise frequency, although residual confounding from unmeasured or imperfectly measured variables cannot be excluded. The covariate-adjusted regression results are presented in Table 5.

**Table 5:** Prediction of main variables by group membership after controlling baseline exercise frequency.

Outcome	Predictor	*B*	SE	beta	*t*	*p*	*R2*
Need_Sat	Group	0.886	0.065	0.725	13.666	< 0.001	0.324
Need_Fru	Group	−1.080	0.069	−0.783	−15.708	< 0.001	0.404
Need_Comp	Group	1.966	0.117	0.821	16.776	< 0.001	0.426
MET_total	Group	3,579.622	146.581	0.959	24.421	< 0.001	0.630
log_MET_total	Group	1.766	0.138	0.686	12.776	< 0.001	0.309
Persist	Group	1.152	0.093	0.675	12.334	< 0.001	0.281
PE_Perf	Group	1.654	0.083	0.874	19.976	< 0.001	0.541
Change	Group	0.803	0.098	0.485	8.176	< 0.001	0.155
SittingMin	Group	−69.388	17.114	−0.256	−4.055	< 0.001	0.041

Group: 0 = control group, 1 = platform-supported group. All models controlled BaseEx. *R2* represents the explained variance of each corresponding model.

### 3.5 Correlation analysis

Correlation analysis showed that Need_Comp was significantly associated with all major physical activity outcomes. Need_Comp was positively correlated with MET_total, log_MET_total, perceived exercise persistence, self-rated PE performance, and perceived change in exercise frequency, and negatively correlated with daily sitting time. The full correlation matrix is provided in Supplementary Table 2.

### 3.6 Cross-sectional indirect associations through need satisfaction and need frustration

Because need satisfaction and need frustration are related but distinct psychological constructs, Need_Sat and Need_Fru were specified as the main parallel psychological variables in the indirect-association models. Results showed that Group was positively associated with Need_Sat (*B* = 0.900, *p* < 0.001) and negatively associated with Need_Fru (*B* = −1.124, *p* < 0.001).

Need_Sat and Need_Fru showed different indirect-association patterns across outcomes. For log_MET_total, the indirect association through Need_Fru was statistically detectable, whereas the indirect association through Need_Sat was not, suggesting that the association with physical activity level was mainly reflected in a reduced need frustration pathway. For perceived exercise persistence and self-rated PE performance, both Need_Sat and Need_Fru showed statistically detectable indirect associations, suggesting a dual pathway of enhanced positive experience and reduced negative psychological barriers. For perceived change in exercise frequency, the Need_Sat indirect association was statistically detectable, whereas the Need_Fru pathway was near the significance boundary, indicating that this outcome was mainly associated with need satisfaction. For daily sitting time, single indirect paths were not consistently detectable, but the total indirect association was detectable; thus, this supplementary outcome should be interpreted cautiously.

Overall, the indirect-association models supported H2: increased need satisfaction and/or reduced need frustration showed statistical indirect associations between platform-supported conditions and physical activity outcomes. These indirect associations should be read as SDT-consistent pathway evidence rather than as strict causal mediation because the psychological variables and outcomes were measured at the same post-intervention time point and complete pretest-posttest measurements were not available. The estimated cross-sectional indirect associations and bootstrap confidence intervals are presented in Table 6.

**Table 6:** Cross-sectional indirect associations through need satisfaction and need frustration.

Outcome	Indirect via Need_Sat	95% CI	Indirect via Need_Fru	95% CI	Total indirect	95% CI
log_MET_total	0.238	[−0.089, 0.535]	0.571	[0.220, 1.084]	0.810	[0.496, 1.215]
Persist	0.440	[0.271, 0.628]	0.306	[0.118, 0.521]	0.746	[0.554, 0.953]
PE_Perf	0.301	[0.122, 0.474]	0.444	[0.229, 0.699]	0.745	[0.560, 0.944]
Change	0.380	[0.215, 0.563]	0.192	[−0.001, 0.397]	0.571	[0.376, 0.785]
SittingMin	−14.426	[−51.967, 17.161]	−38.978	[−77.849, 4.809]	−53.404	[−91.837, −14.169]
MET_total	480.794	[215.714, 777.930]	464.773	[83.824, 887.388]	945.567	[632.088, 1,314.988]

Group: 0 = control group, 1 = platform-supported group. Models controlled BaseEx, gender, grade, and academic major. Indirect associations were tested using 5,000 bootstrap samples. log_MET_total was the primary physical activity outcome, and MET_total was presented as a supplementary result. These models should not be interpreted as causal mediation models because mediators and outcomes were measured at the same post-intervention time point.

### 3.7 Supplementary mediation model using the composite psychological need index

To examine whether the overall psychological need state provided consistent evidence, Need_Comp was further tested as a supplementary psychological need variable. Results showed that Group was positively associated with Need_Comp (*B* = 2.025, *p* < 0.001), and Need_Comp showed significant cross-sectional indirect associations between Group and log_MET_total, perceived exercise persistence, self-rated PE performance, perceived change in exercise frequency, MET_total, and daily sitting time. Full coefficients are provided in Supplementary Table 3.

### 3.8 Robustness analyses

Robustness analyses further supported the direction of the main findings. When log_MET_total was used instead of raw MET_total, the platform-supported group remained significantly higher than the control group. Spearman correlation showed that Need_Comp remained significantly associated with MET_total. After 1% and 99% winsorization of MET_total, both the group difference and the indirect-association result remained statistically detectable. IPAQ completeness checks showed that all 393 participants had complete physical activity data. Detailed robustness results are provided in Supplementary Table 4.

To address multiple comparisons, Benjamini-Hochberg FDR correction was applied separately within the psychological-need family and the physical activity/self-reported outcome family. After correction, all nine psychological-need comparisons and all six physical activity/self-reported outcome comparisons remained statistically detectable, indicating that the reported post-intervention group differences were not solely attributable to unadjusted multiple testing.

Stabilized IPTW sensitivity analyses were also conducted to address observed baseline imbalance. The propensity score model included gender, grade, academic major, and BaseEx. Weighted models produced estimates that were directionally consistent with the main models across all eight primary outcomes. Similar patterns were observed after 1st/99th percentile trimming of extreme weights. However, residual covariate imbalance and extreme weights remained; therefore, these analyses were interpreted as sensitivity evidence for measured baseline covariates rather than causal adjustment for all sources of selection bias.

### 3.9 Data quality auditing and class-clustered robustness

To address potential data quality and class-clustering issues, a full-sample data quality audit was conducted. The audit examined long-string identical responses, extreme contradictions between need satisfaction and need frustration, ceiling patterns in self-reported outcomes, IPAQ extreme values, MET_total outliers, and inconsistent response combinations. In the full sample, 363 responses were classified as clean (92.4%), 24 as minor concern (6.1%), five as moderate concern (1.3%), and one as serious concern (0.3%). Removing moderate and serious concern cases did not change the direction or significance of the main results. Detailed audit information is provided in Supplementary Table 5.

Class-clustered robust standard errors and wild cluster bootstrap tests were then used as sensitivity analyses. After controlling BaseEx, gender, grade, and academic major, Group remained a significant predictor of the main psychological and outcome variables. Because only eight intact classes were included, these results should be treated as supplementary robustness evidence rather than the sole basis for inference. Details are provided in Supplementary Table 5.

## 4 Discussion

### 4.1 From a technological tool to a need-supportive physical activity environment

Guided by SDT, this study examined post-intervention differences in psychological need states and physical activity outcomes under platform-supported conditions in a real university PE course. Overall, the platform-supported group reported higher need satisfaction, lower need frustration, a more favorable composite psychological need state, and more favorable self-reported physical activity outcomes. These findings suggest that the value of a digital-intelligent sports platform should not be reduced to adding an online tool or updating a management method. Rather, platform-supported PE may be understood as a motivational environment in which task access, perceived progress, feedback opportunities, and peer connection are organized differently from regular PE instruction.

In regular PE instruction, teacher guidance is concentrated mainly in the classroom, whereas students‘ extracurricular practice often lacks clear tasks, timely feedback, peer organization, and process records. Through course review, training suggestions, match invitations, ranking feedback, and social interaction, the digital-intelligent sports platform provided additional opportunities for students to encounter sport tasks and peer contexts beyond classroom time (Papastergiou et al., 2021). The higher need satisfaction and lower need frustration in the platform-supported group suggest that digital platforms may be associated with more favorable physical activity outcomes when they are experienced as autonomy-, competence-, and relatedness-supportive conditions. However, given the non-randomized design and absence of complete pretests, these differences should not be interpreted as definitive evidence that the platform caused changes in students' psychological states or physical activity.

### 4.2 Dual-path interpretation of need satisfaction and need frustration

This study treated Need_Sat and Need_Fru as the main parallel psychological variables rather than relying solely on a composite psychological need index. This approach aligns with the SDT position that need satisfaction and need frustration are not simply opposite ends of a single continuum, but related and distinct psychological experiences (Howard et al., 2025). The cross-sectional indirect-association results showed that increased need satisfaction and reduced need frustration were associated with different SDT-consistent pathways across outcomes.

For perceived exercise persistence and self-rated PE performance, both Need_Sat and Need_Fru showed statistically detectable indirect associations, suggesting that students under platform-supported conditions reported stronger positive experiences while also reporting fewer negative psychological barriers. For log_MET_total, the Need_Fru pathway was more prominent than the Need_Sat pathway. This pattern suggests that, in university PE contexts, higher self-reported physical activity may be associated not only with making exercise more attractive, but also with reducing psychological barriers such as feeling incapable, pressured, uncertain about how to practice, or disconnected from participation opportunities. Platform functions such as revisitable movement cues, structured training prompts, match organization, and visible progress records may be consistent with reducing these barriers by making participation more understandable and socially organized. Nevertheless, because mediators and outcomes were measured at the same time point, these results should be interpreted as cross-sectional pathway-consistent evidence rather than evidence of a longitudinal psychological mechanism.

### 4.3 Platform-supported changes in PE support

The platform should not be interpreted as a test of a single AI algorithm. It represents a platform-supported PE environment in which course review, training plans, intelligent question-answering, competition records, and peer interaction reorganized the support available to students beyond the classroom. This framing is important because the study tested the educational environment created by platform use rather than the isolated effect of any single technical module.

This reorganization is relevant for PE because sport learning is embodied and situated. In table tennis, students must coordinate posture, rhythm, footwork, timing, placement judgment, and tactical decisions. Regular classroom instruction provides direct guidance, but it is difficult for a single instructor to support every student's extracurricular practice. Platform-based course review, movement prompts, training tasks, and feedback can therefore extend classroom guidance into students' practice contexts (Hsia et al., 2023).

Interactive technologies may further extend such support by translating sport knowledge and training principles into understandable feedback. However, the present study did not test multimodal diagnosis or a specific algorithmic component. Future research should examine whether distinct platform functions, such as video-based action feedback or adaptive training recommendations, make independent contributions to need satisfaction, need frustration, and physical activity outcomes.

### 4.4 Mobile media, embodied participation, and reconnecting university students with physical activity

University students show relatively high sedentary levels, and mobile media are deeply embedded in their everyday lives (Castro et al., 2020). The platform may have made physical activity more accessible by using a familiar mobile entry point for task understanding, practice organization, competition participation, and peer interaction.

In this sense, mobile access did not replace embodied practice; it helped organize practice before, during, and after participation. Nevertheless, platform design must avoid turning PE into additional screen work or external surveillance. Its educational value depends on whether technology directs students toward movement and supports autonomy, competence, and relatedness.

### 4.5 Result strength, self-report inflation, and interpretive boundaries

Several between-group differences in this study were large, particularly for self-rated PE performance, need frustration, composite psychological need state, and physical activity-related outcomes. These estimates require cautious interpretation. Because the design lacked individual random assignment and complete pretest measures for the primary psychological and outcome variables, Cohen's d values should be read as descriptive standardized post-intervention group differences rather than treatment-effect estimates. Large differences within 8 weeks may partly reflect the platform-supported environment, but they may also reflect baseline nonequivalence, regression to the mean, novelty effects, expectancy effects, demand characteristics, social desirability, and measurement-related inflation (González-Cutre et al., 2016). The possibility of regression to the mean is especially relevant because baseline exercise frequency was lower in the platform-supported group than in the control group. Although BaseEx was statistically controlled, this adjustment cannot directly estimate individual-level change or rule out unmeasured confounding.

Similar studies suggest that digital or gamified physical activity interventions can produce relatively strong participation and psychological responses in the short term, but comparisons with stronger designs should be made carefully. Liang and Wang (2026) used a 10-week quasi-experimental design in app-supported college PE and combined objective pre-post indicators with post-intervention psychological measurement. Hsia et al. (2025) developed an intelligent tutoring and gamified feedback system based on SDT in university PE and reported positive effects on sport skill performance, learning engagement, and autonomy, competence, and relatedness needs. Yang et al. (2026) used a randomized controlled trial and found large effects on average daily steps, daily MVPA, and weekly MET-min. These studies provide relevant context for digital and gamified PE interventions, but their stronger or different designs should not be treated as directly equivalent to the present intact-class post-intervention quasi-experiment.

The supplementary analyses added during revision provide statistical reassurance but do not remove the main design limitations. After Benjamini-Hochberg FDR correction, all psychological-need comparisons and all physical activity/self-reported outcome comparisons remained statistically detectable. Stabilized IPTW analyses based on gender, grade, academic major, and baseline exercise frequency produced directionally consistent estimates across the primary outcomes, and the direction of findings remained consistent after 1st/99th percentile trimming of extreme weights. However, residual covariate imbalance and extreme weights remained, and IPTW can address only measured baseline covariates. Therefore, these results should be interpreted as sensitivity evidence rather than as causal proof.

The self-report nature of the outcome measures further limits interpretation. IPAQ-based physical activity estimates may overestimate actual activity, and self-rated PE performance is not equivalent to objective PE grades or independently assessed motor performance. Harman's single-factor test showed that the first unrotated factor explained 44.40% of the variance, suggesting that common-method variance could not be ruled out. Future research should verify these findings using objective activity monitors, standardized PE performance tests, platform backend logs, independent teaching-fidelity observation, and multi-time-point psychological measurement.

### 4.6 Practical boundaries of platform implementation

Implementing digital-intelligent sports platforms in university PE is not simply a matter of adding an online tool. It involves teachers‘ work patterns, students' participation burden, class time allocation, institutional resources, data governance, and privacy protection. Platforms may reduce fragmented organization and record-keeping, but they also require teacher training, rule explanation, and ongoing guidance.

For students, platforms should serve physical practice rather than create additional reporting tasks or screen burden. If platform use is experienced as a new check-in requirement or extra homework, its need-supportive function may be weakened. Platform design should therefore reduce operational complexity and embed review, training advice, and competition organization into authentic sport participation.

Finally, sustainable implementation depends on development and maintenance capacity, teacher digital literacy, facility conditions, and clear rules for the use of student activity and learning data. These practical conditions should be treated as part of platform-supported PE, not as issues external to the intervention itself.

### 4.7 Limitations and future research

Several limitations should be acknowledged. First, this study adopted an intact-class quasi-experimental design, and students were not randomly assigned at the individual level. Although the two groups were consistent in instructor, sport project, venue, and assessment method, and although baseline exercise frequency and demographic variables were controlled, selection bias arising from intact-class grouping could not be fully excluded. The IPTW sensitivity analysis addressed only observed baseline covariates and cannot eliminate unmeasured confounding.

Second, the groups differed significantly at baseline in gender distribution, academic major, and baseline exercise frequency. Although baseline exercise frequency was lower in the platform-supported group, which makes a simple advantage in prior exercise foundation unlikely, the imbalance still weakens internal validity. Statistical adjustment, FDR correction, class-clustered robust standard errors, wild cluster bootstrap tests, and IPTW analyses provide supplementary sensitivity evidence, but they cannot substitute for random assignment or full pretest-posttest measurement. In addition, class days were not identical across groups: the platform-supported classes were mainly scheduled on Mondays and Tuesdays, whereas control classes were mainly scheduled on Thursdays and Fridays. Different weekdays may involve differences in fatigue, academic pressure, emotional state, and extracurricular arrangements, which may influence PE participation. Future studies should use same-day, same-period, or crossover scheduling when possible to reduce potential time-related confounding.

Third, although all classes were taught by the same instructor, no third-party classroom observer or fidelity assessment was included. Whether the instructor's guidance attitude, feedback frequency, and classroom attention were fully equivalent across groups could not be verified with existing data. Future studies should introduce independent observers, classroom video coding, or teaching fidelity scales to examine implementation fairness and consistency across groups.

Fourth, this study lacked complete pretest-posttest measures. Except for baseline exercise frequency, the primary psychological and outcome variables were measured mainly after the intervention. Therefore, individual-level change processes could not be strictly identified, and regression to the mean cannot be ruled out. The indirect-association findings should be interpreted as statistical indirect associations and potential psychological pathways rather than strict causal mediation mechanisms. Future research should adopt multi-time-point designs, including pre-intervention, mid-intervention, post-intervention, and delayed follow-up measurements, to strengthen temporal ordering and mechanism interpretation.

Fifth, several outcomes were self-reported, including physical activity level, perceived exercise persistence, perceived change in exercise frequency, and self-rated PE performance. Although log transformation, winsorization, FDR correction, IPTW, and sensitivity analyses were used, self-report measures may still be affected by social desirability, recall bias, demand characteristics, expectancy effects, and common-method variance. Future research should combine objective PE grades, physical fitness tests, wearable device records, platform backend logs, and competition participation records to build a more complete evidence chain.

Sixth, individual-level platform use dose could not be exported from the platform backend. Therefore, this study could not analyze the relationships between the intensity of specific platform functions and students' psychological needs or physical activity outcomes, nor could it identify whether students who used the platform more frequently showed stronger outcomes. Future studies should record login frequency, course review views, intelligent question-answering use, match participation, score uploads, and peer interaction to distinguish the specific roles of different platform modules.

Finally, the study was conducted in a table tennis course with first- and second-year undergraduate students aged 18–21 years. Exact individual age was not collected, which limits demographic reporting and prevents age-adjusted analyses. Whether the findings apply to other sport projects, other universities, different student populations, and older or younger students remains to be tested. Future research may compare open-skill and closed-skill sports, individual and team sports, and novice and higher-level students under platform-supported conditions to clarify the boundaries of platform support.

## 5 Conclusion

Because this study used a non-randomized intact-class quasi-experimental design without complete pretest measures for the primary psychological and outcome variables, the findings should be interpreted cautiously as post-intervention group differences and SDT-consistent associations rather than as evidence of causal intervention effects. Within these boundaries, the platform-supported condition was associated with higher basic psychological need satisfaction, lower basic psychological need frustration, higher self-reported physical activity level, greater perceived exercise persistence, more favorable perceived change in exercise frequency, higher self-rated PE performance, and lower sitting time. Cross-sectional indirect-association models showed that increased need satisfaction and reduced need frustration displayed SDT-consistent indirect associations between group membership and multiple physical activity outcomes, while the supplementary Need_Comp model provided consistent evidence.

These findings suggest that the educational value of a platform-supported PE environment may lie not merely in exercise recording, task delivery, or teaching management. Rather, such an environment may be most useful when it provides clear practice pathways, actionable feedback, peer interaction, competition organization, process records, and opportunities for visible progress. For PE teachers, the practical implication is not to use digital platforms primarily as surveillance or check-in tools, but to design platform-supported activities that help students understand what to practice, perceive how they are improving, and connect with peers in authentic sport participation. Given the design limitations, these implications should be treated as tentative and should be verified through randomized or better-controlled pretest-posttest studies using objective activity and performance indicators.

## Data Availability

The raw data supporting the conclusions of this article will be made available by the authors, without undue reservation.
