# Supplementary material for: Need-supportive digital sports platform and physical activity outcomes in university physical education: an intact-class quasi-experimental study based on self-determination theory
Source: Front Psychol. 2026 Jul 29;17:1889324. doi: 10.3389/fpsyg.2026.1889324 (PMC13461492; doi:10.3389/fpsyg.2026.1889324)
Supplement: Supplementary file 1 [file Data_Sheet_1.pdf]

Supplementary Material

Supplementary tables for: Need-Supportive Digital Sports Platform and Physical Activity Outcomes: An Intact-Class Quasi-Experimental Study Based on Self-Determination Theory

Supplementary Table S1. Reliability and structural validity of BPNSFS.

Indicator	Result	Interpretation
Cronbach's alpha	0.753-0.879	Acceptable to good internal consistency for subdimensions
McDonald's omega	0.766-0.883	Acceptable to good internal consistency for subdimensions
Need_Sat alpha/omega	0.917 / 0.919	Good
Need_Fru alpha/omega	0.912 / 0.916	Good
chi-square/df	2.270	Acceptable
CFI	0.944	Good
TLI	0.934	Good
RMSEA	0.057	Good
SRMR	0.047	Good

Note. The six-factor BPNSFS model included autonomy satisfaction, autonomy frustration, competence satisfaction, competence frustration, relatedness satisfaction, and relatedness frustration. AVE values for relatedness satisfaction and relatedness frustration were slightly below 0.50, and these dimensions should be interpreted cautiously.

Supplementary Table S2. Correlation matrix of main variables.

Variable	1	2	3	4	5	6	7	8
1. Group	—							
2. BaseEx	-0.620***	—						
3. Need_Sat	0.464***	-0.028	—					
4. Need_Fru	-0.584***	0.164**	-0.696***	—				
5. Need_Comp	0.572***	-0.109*	0.910***	-0.931***	—			
6. MET_total	0.747***	-0.253***	0.605***	-0.655***	0.686***	—		
7. Persist	0.420***	-0.007	0.611***	-0.572***	0.641***	0.584***	—	
8. PE_Perf	0.703***	-0.266***	0.643***	-0.712***	0.738***	0.743***	0.573***	—
9. Change	0.363***	-0.104*	0.475***	-0.442***	0.496***	0.461***	0.488***	0.422***
10. SittingMin	-0.141**	-0.026	-0.222***	0.241***	-0.252***	-0.155**	-0.214***	-0.210***

Note. Values are Pearson correlation coefficients. Group: 0 = control group, 1 = platform-supported group. *p < 0.05, **p < 0.01, ***p < 0.001.

Supplementary Table S3. Supplementary mediation model using the composite psychological need index.

Outcome	a	b	cprime	Indirect effect	95% CI	R2(Y)	N
log_MET_total	2.025***	0.391***	0.999***	0.791	[0.472, 1.188]	0.388	393
Persist	2.025***	0.376***	0.350**	0.762	[0.574, 0.963]	0.445	393
PE_Perf	2.025***	0.366***	0.994***	0.740	[0.562, 0.928]	0.672	393
Change	2.025***	0.291***	0.170	0.590	[0.398, 0.798]	0.276	393
SittingMin	2.025***	-25.686***	-5.765	-52.008	[-90.293, -13.328]	0.090	393
MET_total	2.025***	471.455***	2691.413***	954.600	[635.988, 1295.891]	0.685	393

Note. a indicates Group -> Need_Comp, b indicates Need_Comp -> outcome, and cprime indicates the direct effect of Group -> outcome after controlling Need_Comp. All models controlled BaseEx, gender, grade, and academic major. Indirect effects were tested using 5,000 bootstrap samples. **p < 0.01, ***p < 0.001.

Supplementary Table S4. Robustness analysis results.

Analysis	Estimate	p	Description
Group difference using log_MET_total	1.307	<0.001	Welch t test
Spearman: Need_Comp with MET_total	0.714	<0.001	N = 393
Group difference after winsorizing MET_total	2790.437	<0.001	1st/99th percentile winsorization
Mediation effect after winsorizing MET_total	926.511	—	95% CI [634.893, 1247.599]
IPAQ completeness check	393/393	—	No incomplete IPAQ cases in current data

Note. Winsorization was used to reduce the potential influence of extreme values on MET_total. The mediation effect was evaluated using bootstrap confidence intervals.

Supplementary Table S5. Data quality auditing and class-clustered robustness analysis.

Analysis	Main result	Interpretation
Full-sample data quality	clean = 363/393; serious concern = 1/393	No large-scale invalid responding
Platform group data quality	clean = 186/202; serious concern = 0	Overall high quality in platform group
Control group data quality	clean = 177/191; serious concern = 1	Overall high quality in control group
Sensitivity after deleting suspicious cases	Direction and significance of main results unchanged	Findings not driven by suspicious responses
Cluster-robust SE	All main results remained significant	Supports result stability
Wild cluster bootstrap	Main result direction supported	Supplementary evidence with eight intact classes

Note. Suspicious cases refer to moderate concern and serious concern cases. Class-clustered robustness analyses controlled BaseEx, gender, grade, and academic major. Because only eight intact classes were included, clustered results were treated as supplementary sensitivity evidence.

Supplementary Table S6. FDR-corrected post-intervention group differences

Family	Variable	Control M (SD)	Platform-supported M (SD)	Mean difference	Welch t	df	Raw p	FDR-adjusted p	Cohen's d
psychological needs	Need_Sat	3.83 (0.72)	4.39 (0.29)	0.566	10.140	245.6	< .001	< .001	1.045
psychological needs	Need_Fru	2.31 (0.77)	1.51 (0.23)	-0.805	-13.882	222.1	< .001	< .001	-1.434
psychological needs	Need_Comp	1.52 (1.36)	2.89 (0.38)	1.372	13.479	218.4	< .001	< .001	1.393
psychological needs	Auto_Sat	3.88 (0.83)	4.39 (0.35)	0.509	7.828	251.2	< .001	< .001	0.806
psychological needs	Auto_Fru	2.11 (0.95)	1.52 (0.30)	-0.591	-8.191	224.9	< .001	< .001	-0.846
psychological needs	Comp_Sat	3.73 (0.83)	4.39 (0.37)	0.660	10.123	259.8	< .001	< .001	1.041
psychological needs	Comp_Fru	2.95 (0.94)	1.51 (0.31)	-1.442	-20.149	228.7	< .001	< .001	-2.080
psychological needs	Rel_Sat	3.88 (0.73)	4.41 (0.33)	0.530	9.190	259.2	< .001	< .001	0.945
psychological needs	Rel_Fru	1.87 (0.78)	1.49 (0.31)	-0.383	-6.369	245.3	< .001	< .001	-0.656
outcomes	MET_total	2120.21 (1547.25)	4908.24 (862.15)	2788.023	21.896	294.0	< .001	< .001	2.243
outcomes	log_MET_total	7.18 (1.58)	8.48 (0.18)	1.307	11.342	194.8	< .001	< .001	1.176
outcomes	Persist	3.39 (0.97)	4.11 (0.53)	0.716	9.012	288.8	< .001	< .001	0.924
outcomes	PE_Perf	3.13 (0.81)	4.46 (0.52)	1.330	19.303	321.3	< .001	< .001	1.971
outcomes	Change	3.52 (0.97)	4.12 (0.52)	0.600	7.575	286.1	< .001	< .001	0.777
outcomes	SittingMin	413.01 (183.29)	374.78 (57.57)	-38.233	-2.757	225.3	0.006	0.006	-0.285

Note. FDR = false discovery rate. Benjamini-Hochberg correction was applied separately within the psychological-need family and the physical activity/self-reported outcome family. The comparisons are descriptive post-intervention group differences and should not be interpreted as causal treatment effects.

Supplementary Table S7. Propensity score balance and stabilized IPTW weight diagnostics

Panel A. Covariate balance before and after stabilized IPTW

Covariate	Level	Type	Unweighted control	Unweighted platform	SMD before weighting	Weighted control	Weighted platform	SMD after weighting
BaseEx		continuous	3.246	2.035	-1.579	2.538	2.938	0.345
gender	female	categorical level	0.257	0.495	0.508	0.460	0.371	-0.182
gender	male	categorical level	0.743	0.505	-0.508	0.540	0.629	0.182
grade	1	categorical level	0.340	0.441	0.207	0.416	0.585	0.343
grade	2	categorical level	0.660	0.559	-0.207	0.584	0.415	-0.343
major	education_arts_pe	categorical level	0.131	0.317	0.458	0.162	0.308	0.350
major	humanities	categorical level	0.079	0.238	0.447	0.154	0.207	0.138
major	social_science	categorical level	0.126	0.282	0.396	0.241	0.167	-0.184
major	stem_medicine	categorical level	0.665	0.163	-1.183	0.443	0.318	-0.260

Panel B. Stabilized IPTW weight distribution

Statistic	Value
N used in PS model	393.000
min	0.487
max	27.639
mean	1.068
SD	2.294
1st percentile	0.487
99th percentile	9.665
extreme weights flag	1.000

Note. IPTW = inverse probability of treatment weighting; SMD = standardized mean difference. The propensity score model included gender, grade, academic major, and baseline exercise frequency. The age variable was not available in the questionnaire dataset and therefore could not be included. Extreme weights were detected; consequently, 1st/99th percentile trimmed IPTW results are also reported in Supplementary Table S8. Residual imbalance remained after weighting, so these analyses should be interpreted as sensitivity analyses for observed covariates rather than causal adjustment.

Supplementary Table S8. IPTW-weighted sensitivity analyses

Outcome	Weight set	N	B	Robust SE	95% CI	p	Direction consistency
Need_Sat	stabilized IPTW	393	0.932	0.171	[0.596, 1.267]	< .001	consistent
Need_Fru	stabilized IPTW	393	-1.319	0.177	[-1.668, -0.970]	< .001	consistent
Need_Comp	stabilized IPTW	393	2.251	0.330	[1.601, 2.900]	< .001	consistent
log_MET_total	stabilized IPTW	393	1.810	0.193	[1.430, 2.191]	< .001	consistent
Persist	stabilized IPTW	393	1.339	0.275	[0.799, 1.880]	< .001	consistent
PE_Perf	stabilized IPTW	393	1.828	0.241	[1.355, 2.302]	< .001	consistent
Change	stabilized IPTW	393	0.739	0.110	[0.522, 0.956]	< .001	consistent
SittingMin	stabilized IPTW	393	-79.760	24.873	[-128.660, -30.859]	0.001	consistent
Need_Sat	1st/99th percentile trimmed IPTW	393	0.866	0.136	[0.599, 1.133]	< .001	consistent
Need_Fru	1st/99th percentile trimmed IPTW	393	-1.209	0.124	[-1.452, -0.965]	< .001	consistent
Need_Comp	1st/99th percentile trimmed IPTW	393	2.075	0.236	[1.610, 2.540]	< .001	consistent
log_MET_total	1st/99th percentile trimmed IPTW	393	1.733	0.170	[1.398, 2.067]	< .001	consistent
Persist	1st/99th percentile trimmed IPTW	393	1.165	0.170	[0.830, 1.499]	< .001	consistent
PE_Perf	1st/99th percentile trimmed IPTW	393	1.707	0.146	[1.420, 1.994]	< .001	consistent
Change	1st/99th percentile trimmed IPTW	393	0.733	0.111	[0.515, 0.950]	< .001	consistent
SittingMin	1st/99th percentile trimmed IPTW	393	-67.195	19.165	[-104.874, -29.516]	< .001	consistent

Note. Weighted linear models estimated the association between group status and each outcome using stabilized IPTW weights and robust standard errors. Trimmed results used 1st/99th percentile weight trimming because extreme weights were identified. These analyses address only observed baseline covariates and cannot eliminate unmeasured confounding or compensate for the absence of complete pretest measures.

Supplementary Note. Data limitations relevant to interpretation

- Participant age was not individually collected in the questionnaire dataset. Based on course enrollment, participants were first- and second-year university students aged approximately 18-21 years.
- No objective individual-level platform usage indicators were found in the available questionnaire exports; therefore, dose-response associations between actual platform engagement and outcomes could not be examined.
- Complete pretest measures for the primary psychological and outcome variables were not available; therefore, individual-level change and longitudinal causal mediation could not be estimated.
- Harman's single-factor test showed that the first unrotated component explained 44.40% of the variance across 24 BPNSFS item-level variables. This result does not rule out common-method variance and should be interpreted as a limitation of the self-report design.
